# The MeCP2E1/E2-BDNF-*miR132* Homeostasis Regulatory Network Is Region-Dependent in the Human Brain and Is Impaired in Rett Syndrome Patients

**DOI:** 10.3389/fcell.2020.00763

**Published:** 2020-08-21

**Authors:** Shervin Pejhan, Marc R. Del Bigio, Mojgan Rastegar

**Affiliations:** ^1^Regenerative Medicine Program, Department of Biochemistry and Medical Genetics, Max Rady College of Medicine, Rady Faculty of Health Sciences, University of Manitoba, Winnipeg, MB, Canada; ^2^Department of Pathology, Max Rady College of Medicine, Rady Faculty of Health Sciences, University of Manitoba, Winnipeg, MB, Canada

**Keywords:** Rett Syndrome, MeCP2 isoforms, MeCP2E1, MeCP2E2, BDNF, *miR132*, Epigenetics, DNA methylation

## Abstract

Rett Syndrome (RTT) is a rare and progressive neurodevelopmental disorder that is caused by *de novo* mutations in the X-linked Methyl CpG binding protein 2 (*MECP2*) gene and is subjected to X-chromosome inactivation. RTT is commonly associated with neurological regression, autistic features, motor control impairment, seizures, loss of speech and purposeful hand movements, mainly affecting females. Different animal and cellular model systems have tremendously contributed to our current knowledge about MeCP2 and RTT. However, the majority of these findings remain unexamined in the brain of RTT patients. Based on previous studies in rodent brains, the highly conserved neuronal microRNA “*miR132*” was suggested to be an inhibitor of MeCP2 expression. The neuronal *miR132* itself is induced by Brain Derived Neurotrophic Factor (BDNF), a neurotransmitter modulator, which in turn is controlled by MeCP2. This makes the basis of the *MECP2-BDNF-miR132* feedback regulatory loop in the brain. Here, we studied the components of this feedback regulatory network in humans, and its possible impairment in the brain of RTT patients. In this regard, we evaluated the transcript and protein levels of *MECP2*/MeCP2E1 and E2 isoforms, *BDNF*/BDNF, and *miR132* (both *3p* and *5p* strands) by real time RT-PCR, Western blot, and ELISA in four different regions of the human RTT brains and their age-, post-mortem delay-, and sex-matched controls. The transcript level of the studied elements was significantly compromised in RTT patients, even though the change was not identical in different parts of the brain. Our data indicates that MeCP2E1/E2-BDNF protein levels did not follow their corresponding transcript trends. Correlational studies suggested that the *MECP2E1/E2-BDNF-miR132* homeostasis regulation might not be similarly controlled in different parts of the human brain. Despite challenges in evaluating autopsy samples in rare diseases, our findings would help to shed some light on RTT pathobiology, and obscurities caused by limited studies on MeCP2 regulation in the human brain.

## Introduction

The X-linked Methyl CpG binding protein 2 (*MECP2*) gene encodes for an important nuclear factor that binds to methylated DNA. MeCP2 protein is the prototype member of the methyl binding proteins, which was discovered almost three decades ago (Lewis et al., 1992). MeCP2 is a multi-functional epigenetic factor and its proper homeostasis regulation is critical for normal brain development (Delcuve et al., 2009; Zachariah and Rastegar, 2012; Rastegar, 2017b; Tillotson and Bird, 2019). Mutations in the *MECP2* gene located in *Xq28* are the underlying cause of over 90% of typical and a portion of atypical cases of Rett Syndrome (RTT) (Neul et al., 2008; Liyanage and Rastegar, 2014; Chahil and Bollu, 2020), a severe neurodevelopmental disorder that is primarily detected in females (Amir et al., 1999). The vast majority of these mutations happen *de novo* and mostly in the paternal germline (Girard et al., 2001; Trappe et al., 2001). There are also rare familial cases of RTT that may happen due to skewing of X chromosome inactivation (XCI) in the mother, who can pass *MECP2* mutation(s) on to male and female offspring as an asymptomatic carrier (Villard et al., 2000). The pattern of XCI has been studied in RTT, however, the results are not fully conclusive (Xinhua et al., 2008). Although the majority of classical cases of RTT have balanced XCI pattern in the brain tissue (Zoghbi et al., 1990; Shahbazian et al., 2002a, b), non-random XCI or skewed pattern, has also been reported in several cases (Renieri et al., 2003).

In mice and humans, the *Mecp2*/*MECP2* gene consists of 4 coding exons separated by 3 introns (Liyanage and Rastegar, 2014). Although several protein-coding and non-coding *MECP2* transcripts have been identified or predicted (Singh et al., 2008), the two most-studied splice variants of MeCP2 are known as MeCP2E1 and MeCP2E2 (Kriaucionis and Bird, 2004; Mnatzakanian et al., 2004). Accordingly, MeCP2E1 and E2 isoform-specific expression, regulation, function, and clinical relevance to RTT have been the focus of several research studies (Rastegar et al., 2009; Itoh et al., 2012; Kerr et al., 2012; Zachariah et al., 2012; Liyanage et al., 2013, 2019; Olson et al., 2014; Yasui et al., 2014; Sheikh et al., 2017; Vogel Ciernia et al., 2018; Martinez de Paz et al., 2019; Takeguchi et al., 2020). Information about other *MECP2* transcripts and their tissue- and cell-type specific expression could be obtained through RNA sequencing or data mining of related publically available data repositories. Among the known *MECP2* protein-coding transcripts, MeCP2E2 was the original protein variant that was discovered in 1992 (also referred to as MeCP2β or MeCP2A) (Lewis et al., 1992). In 2004, another splice variant of MeCP2 was identified, currently known as MeCP2E1 (also called MeCP2α or MeCP2B) (Kriaucionis and Bird, 2004; Mnatzakanian et al., 2004). MeCP2E1 is encoded by exons 1, 3, and 4; while MeCP2E2 is encoded by exons 2, 3, and 4. The two MeCP2E1 (498-aa) and E2 (486-aa) isoforms fully share MeCP2 protein domains and only differ in their short N-terminal regions that consist of 21-aa exclusive to the MeCP2E1, and 9-aa specific to MeCP2E2 (Kriaucionis and Bird, 2004; Mnatzakanian et al., 2004; Zachariah et al., 2012; Liyanage and Rastegar, 2014; Olson et al., 2014). The larger MeCP2E1 isoform is the dominant protein in the brain with relatively consistent expression throughout different brain regions of the adult mice, and earlier developmental onset of expression in the brain (Olson et al., 2014). Interestingly, E1-deficiency in mice mimics similar phenotype as mice lacking the whole *Mecp2* gene (Yasui et al., 2014), suggesting its importance in RTT pathobiology. In support of this conclusion, E2-deficient mice do not exhibit RTT-associated symptoms (Itoh et al., 2012), suggesting that E2 may not have direct relevance to RTT.

In the brain, compromised MeCP2 expression and/or function is associated with impaired brain function associated with autism, RTT, and *MECP2* duplication Syndrome (MDS) (Liyanage and Rastegar, 2014). Our previous studies have highlighted the cell type-specific role of DNA methylation, and murine strain in *Mecp2*/MeCP2E1/E2 regulation (Liyanage et al., 2013, 2015, 2019; Olson et al., 2014; Xu et al., 2019; Amiri et al., 2020). Studies have also shown that in rodent brain/brain cells MeCP2 homeostasis is controlled *via* a regulatory feedback loop consisting of MeCP2, brain-derived neurotrophic factor (BDNF), and a neuronal-specific microRNA named *miR132* (Klein et al., 2007). The monogenic character of RTT, mainly caused by mutations in the *MECP2* gene has prompted several RTT animal, and development of cellular (Rastegar et al., 2009; Marchetto et al., 2010; Yazdani et al., 2012; Ezeonwuka and Rastegar, 2014) model systems to study disease mechanism. However, the brain region-specific expression pattern of the suggested MeCP2-BDNF-*miR132* regulatory network in the human brains is unknown. Furthermore, it is not clear if the components of this homeostasis regulatory network are affected in the human RTT brain, mainly due to the limitation of access to human brain tissues for this rare disorder (1:10,000–15,000 females) (Amir et al., 1999).

Here, we investigated the expression of the suggested MeCP2 homeostasis regulatory network, while studying both MeCP2 isoforms in post-mortem human brain tissues of RTT patients and age-/sex-matched controls. We employed quantitative and semi-quantitative approaches including real time reverse transcriptase-PCR (RT-PCR), ELISA, and Western blot (WB) to study the components of MeCP2E1/E2-BDNF-*miR132* feedback loop at the transcript and protein levels. Our results indicate that *MECP2E1* transcripts are higher than *MECP2E2* in the human frontal cerebrum, hippocampus, amygdala, and cerebellum, and that the latter shows significantly higher levels of *MECP2E1* and *MECP2E2* compared to other tested brain regions. We further show that not only *MECP2E1* and *MECP2E2*, but also *BDNF* transcripts are significantly lower in RTT brains compared to controls. We observed that *miR132-3p* is the dominant strand in all studied human brain regions with significantly lower levels in the cerebellum. Combined correlational analysis of our transcript and protein studies suggests a potential regulatory feedback of *MECP2*/MeCP2-*BDNF*/BDNF in the brain, which might be independent of *miR132* in specific parts of the human brain.

## Materials and Methods

### Ethics Statement

Our research studies on the human brain tissues presented in this manuscript were reviewed and approved by the “University of Manitoba Bannatyne Campus Research Ethics Board” and under an approved Health Research Board protocol #HS20095 (H2016:337).

### Human Brain Tissues

Autopsy brain samples of three female RTT patients were analyzed in this study in parallel to their comparable age- and sex-matched controls. RTT brain samples were from: (A) a 13-year-old female Rett Syndrome patient with the most frequent MeCP2 mutation (T158M). The brain tissues for this patient was received by organ donation, and the related clinical data are already reported (Olson et al., 2018), (B) a 19-year-old female RTT patient (A201V). The brain tissue for this patient was also received through direct organ donation, and (C) the third patient was a 20-year-old female RTT patient (R255X), for whom the brain samples were received from NIH Neurobiobank (NIH # 4516). Tissues from three comparable female control (non-RTT) brain tissues were received from NIH Neurobiobank. Table 1 provides the information about these brain samples. Two patients with T158M and R255X mutations are representatives of the first and third most common RTT-associated point mutations that cause the disease (RettBASE Database) (Krishnaraj et al., 2017). These two mutations (T158M and R255X) affect two of the main protein functional domains, namely the methyl binding domain (involved in DNA binding of MeCP2) and the transcriptional repression domain (involved in protein-protein interactions), respectively (Liyanage and Rastegar, 2014). The third patient with clinical diagnosis of RTT had a polymorphism (A201V) known to be associated with RTT. Based on potential link to disease symptoms such as cognitive, behavioral, and locomotor impairments in RTT patients, four brain regions were selected for our study that included the frontal cerebrum, hippocampus, amygdala, and cerebellum. Hematoxylin and Eosin (H&E) stained slides of the human brain tissue were prepared by paid services from the Health Sciences Center, Pathology Laboratory, Winnipeg, and Department of Human Anatomy and Cell Science, University of Manitoba. Imaging and analysis of the images were done by the authors.

**TABLE 1:** The list of human brain samples used in our study.

**NIH#**	**RTT/Control**	**Age(Years)**	**Sex**	**PMD**
*	T158M	13	F	<6 h
*	A201V	19	F	24 h
4516	R255X	20	F	9 h
5401	Control	19	F	22 h
5646	Control	20	F	23 h
5287	Control	23	F	15 h
¤	Control	14	F	24 h
¤	Control	18	F	48 h

All patients and controls are Caucasian. Two RTT brain samples (T158M and A201V) were donated directly to our lab for research (*), and were coordinated through collaboration with Dr. Victoria Siu (Western University) who obtained proper consent for research, as reported previously (Olson et al., 2018; Pejhan et al., 2020). Two control brain samples for Hematoxylin and Eosin (H&E) staining were provided by the Health Sciences Centre Pathology Laboratory, Winnipeg, MB (¤), coordinated by Dr. Marc Del Bigio. Post-mortem delay (PMD) shows the interval between death and tissue preservation. Samples from NIH NeuroBioBank were obtained by Dr. Mojgan Rastegar and signed Material Transfer Agreement (MTA); F (Female).

### Quantitative Real Time Reverse-Transcriptase Polymerase Chain Reaction (qRT-PCR)

Total RNA was extracted from the frontal cerebrum, hippocampus, amygdala, and cerebellum by using Trizole (Life Technologies Inc., 15596-062) as per manufacturer’s guidelines, and as previously reported (Rastegar et al., 2004; Huang et al., 2005; Kobrossy et al., 2006). The extracted total RNA was treated with DNase I for eliminating traces of possible contamination with genomic DNA, by using Ambion TURBO DNA-free kit (Thermo Fisher Scientific). Total RNA was then processed for cDNA synthesis by Superscript III Reverse Transcriptase (Invitrogen), as described elsewhere (Pejhan et al., 2020). For quantitative RT-PCR, we used a Fast 7500 Real-Time PCR machine (Applied Biosystems) with SYBR Green-based RT-PCR Master Mix reagents (Applied Biosystems). Transcripts levels of *MECP2* isoforms and *BDNF* were assessed using specific primers that are listed in Supplementary Table S1, being normalized to *GAPDH* (*Glyceraldehyde 3-phosphate dehydrogenase)*, as previously described (Barber et al., 2013; Xu et al., 2019). We used Microsoft Excel 2.10 and GraphPad Prism 7 to analyze the results.

### TaqMan Assay for *miR132* Detection

The TaqMan^®^MicroRNA assay kit from Thermo Fisher was used to analyze and quantify *miR132*. First cDNA was reverse transcribed from DNase-treated RNA by TaqMan^®^MicroRNA reverse transcription kit (Thermo Fisher Cat. No. 4366596), as per manufacturer’s protocol and guidelines. The generated cDNA was used to perform RT-PCR reaction for *miR132*-*3p*, *miR132*-*5p*, and *U6* with the TaqMan primers from Thermo Fisher (hsa-*miR132*-*3p*, Cat. No. 000457, hsa-*miR132*-*5p*, Cat. No. 002132, and *U6* snRNA, Cat. No. 001973). We selected *U6* snRNA as an internal miRNA control, as suggested by previous studies (McDermott et al., 2013; Duan et al., 2018). The results for two *miR132* strands were normalized to *U6* snRNA (endogenous control), with the same analysis method as the other studied genes here.

### Western Blot

Nuclear and cytoplasmic protein fractions were isolated from frozen frontal cerebrum, hippocampus, amygdala, and cerebellum samples by NE-PER Extraction Kit (Thermo Scientific, Cat. No. 78833), according to the manufacturer’s instruction and as described previously (Olson et al., 2014, 2018). Western blot experiments were performed with the described protocols in previous reports (Rastegar et al., 2000; Wu et al., 2001; Gordon et al., 2003; Sheikholeslami et al., 2019). As housekeeping protein and loading control, GAPDH was checked on all membranes and the results for each antibody were normalized to GAPDH signals. Validation of GAPDH in the nuclear and cytoplasmic extracts from human RTT and control brain tissues has been also reported recently (Olson et al., 2018). The list of primary and secondary antibodies is provided in Supplementary Table S2. MeCP2E1 and MeCP2E2 antibodies are custom-made and have been previously characterized and reported for detecting endogenous protein isoforms (Zachariah et al., 2012; Liyanage et al., 2013; Olson et al., 2014; Yasui et al., 2014). BDNF antibody was purchased from Abcam (108319).

### Enzyme-Linked Immunosorbent Assay (ELISA)

We used ELISA to quantify BDNF protein in the cytoplasmic protein extracts of the human brain tissues. Human BDNF ELISA kit from Sigma-Aldrich (RAB0026) was used according to the manufacturer’s instructions. For intra and inter-assay coefficients of variation, we used <10% and <12%, respectively, while the sensitivity was determined at 80 pg/ml. Absorbance was measured on SpectraMax M2e plate reader (Molecular Devices), and concentrations were calculated based on a standard curve that was generated by the Softmax Pro 5.3. BDNF levels were calculated as ng/mg of total protein.

### Statistical Analysis

GraphPad Prism software was used for statistical analysis and generating the graphs, as reported (Barber et al., 2013; Liyanage et al., 2015; Sheikholeslami et al., 2019). Statistical significance was determined by one-way ANOVA with multiple pairwise comparisons among different brain regions for transcript assessments and protein quantifications by ELISA. We used Student’s *t*-test to compare controls and RTT samples for these parametric data. Western blot non-parametric results were analyzed by Kruskal-Wallis, and Mann-Whitney tests. Pearson correlation study was used to measure the degree of the relationship between parametric variables, and Spearman’s for non-parametric ones. The significance level was calculated at ^∗^*P* < 0.05, ^∗∗^*P* < 0.01, ^∗∗∗^*P* < 0.001, and ^****^P < 0.0001.

## Results

### MECP2E1 and MECP2E2 Transcripts Are Significantly Lower in RTT Patients Compared to Controls

**FIGURE 1:**
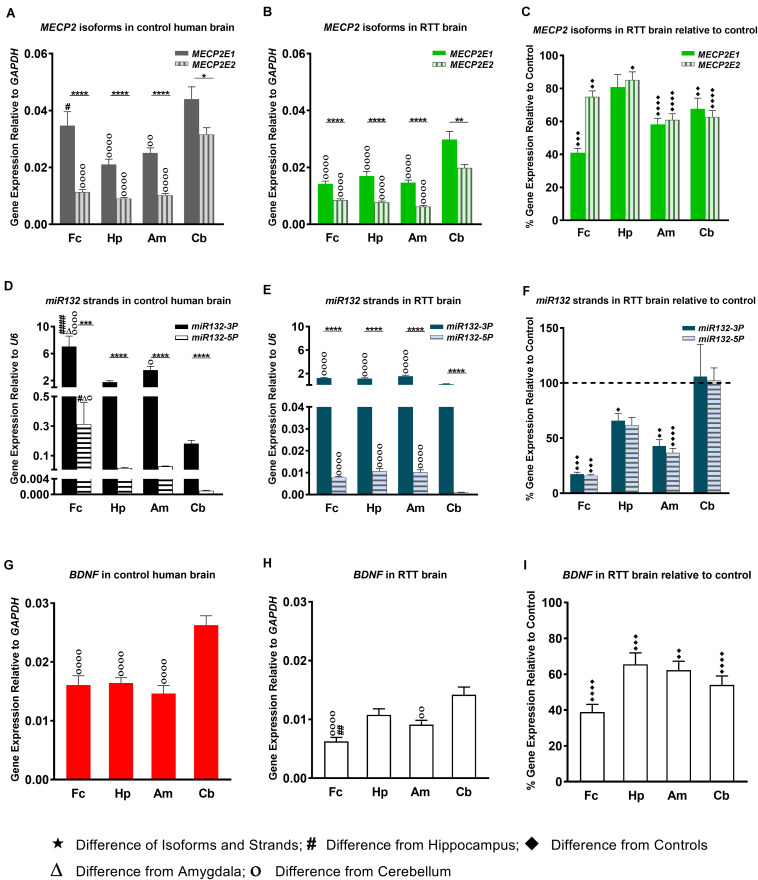
Transcript levels of *MECP2E*1, *MECP2E2*, *BDNF*, and *miR132*. In all studied brain regions of three RTT **(B,E)** and controls **(A,D)**, *MECP2E*1 and *miR132-3P* are significantly higher than *MECP2E2* and *miR132-5P*, respectively. Cerebellum in comparison with the other three brain regions shows the highest level for all the studied transcripts except for *microRNA132* **(A,B,D,E,G,H)**. Rett Syndrome (RTT) patients show lower transcript levels of *MECP2E1*, *MECP2E2*, *BDNF*, *miR132-3P*, and *miR132-5P* in four studied brain regions compared to their age-, and sex- matched controls **(C,F,I)**. Cerebellum has exceptionally similar level of *miRNAs* in RTT and controls **(F)**. Frontal cerebrum (Fc), Hippocampus (Hp), Amygdala (Am), Cerebellum (Cb); *N* = 3 ± Standard Error of the Mean (SEM); One-way ANOVA with multiple pairwise comparisons used among different brain regions. Student’s *t*-test was used to compare controls and RTT samples for each parameter. The significance level was determined at single symbol *P* < 0.05, double symbols *P* < 0.01, triple symbols *P* < 0.001, and quadruple symbols *P* < 0.0001.

In order to study *MECP2E1* and *MECP2E2* transcript levels, we performed qRT-PCR in isolated RNA samples from the frontal cerebrum, hippocampus, amygdala, and cerebellum of RTT patients and age-/sex-matched control brain tissues. Our results indicated that *MECP2E1* transcripts are significantly higher than *MECP2E2* transcripts in all the studied brain regions of the controls and RTT patients (Figures 1A,B). In control brain tissues, both isoforms showed higher transcript levels in the cerebellum compared to other brain regions, with a similar trend in RTT patients. Comparing *MECP2E1* and *MECP2E2* transcript levels in RTT patients versus controls, we observed significantly lower levels of *MECP2E1* and *MECP2E2* isoforms in RTT patients in all four tested brain regions (Figure 1C). The severity of such significantly reduced expression was brain region-dependent, ranging from 20% in the hippocampus to 60% in the frontal cerebrum (Table 2).

**TABLE 2:** Summary of changes at transcript level in the studied element of four brain regions in RTT patients compared to controls.

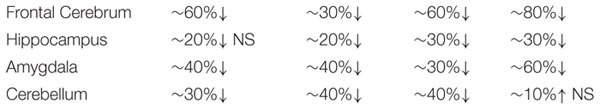

*NS, not statistically significant.*

### Transcript Detection of *miR132* and BDNF in the Brain of RTT Patients and Controls

Next, we aimed to investigate the transcript levels of *miR132* (both *3p* and *5p* strands) in the brain of RTT patients and control tissue samples. Isolated RNA from the selected four brain regions was subjected to TaqMan real time PCR. Our results indicated that between the two strands, *miR132-3p* is the dominant transcript in all tested brain regions of control and RTT brain tissue samples. Despite its significantly lower level, the *miR132-5p* strand still showed a similar pattern in comparison with the *miR132-3p* among different brain regions of RTT and controls (Figures 1D–F). Analysis of these results indicated that compared to controls, RTT brain regions had significantly lower levels of *miR132-3p* in the frontal cerebrum, hippocampus, and amygdala, but they were equal in the cerebellum (Figure 1F). The minimum ratio belonged to RTT frontal cerebrum with less than 18% of the control levels (*P* < 0.001). Amygdala with less than 43% (*P* < 0.01), and hippocampus with less than 66% of control level (*P* < 0.05) were the next two regions.

In order to study the components of MeCP2 homeostasis regulation, next we analyzed *BDNF* transcripts in the control and RTT brain tissues. In control brain samples, *BDNF* transcripts were relatively consistent in the frontal cerebrum, hippocampus, and amygdala. However, *BDNF* transcripts were found to be about 1.5 times higher in the cerebellum compared to the other tested brain regions (*P* < 0.0001) (Figure 1G). Similar analysis of RTT brain tissues showed significantly higher *BDNF* levels in the cerebellum compared to the frontal cerebrum (*P* < 0.0001) and amygdala (*P* < 0.01). Although, *BDNF* transcripts in the cerebellum were still higher compared to hippocampus, the detected change did not reach to any statistical significance (Figure 1H). Next, we compared BDNF transcript levels of control and RTT brain tissues. In all four tested brain regions of RTT samples; *BDNF* transcripts were significantly lower compared to controls (Figure 1I). Such significantly lower levels in RTT brains ranged from 30% reduced *BDNF* levels in the hippocampus (*P* < 0.001) and amygdala (*P* < 0.01) to 60% in the frontal cerebrum (*P* < 0.0001) (Table 2). Taken together, our results indicate that not only *miR132* (both *miR132-3p* and *miR132-5p)* but also *BDNF* transcripts are significantly lower in RTT patients, and that the *miR132-3p* is the dominant microRNA strand in the human brain, at least in the four tested brain regions in this study.

### Expression Analysis of MeCP2 Isoforms in RTT and Control Human Brain Tissues

**FIGURE 2:**
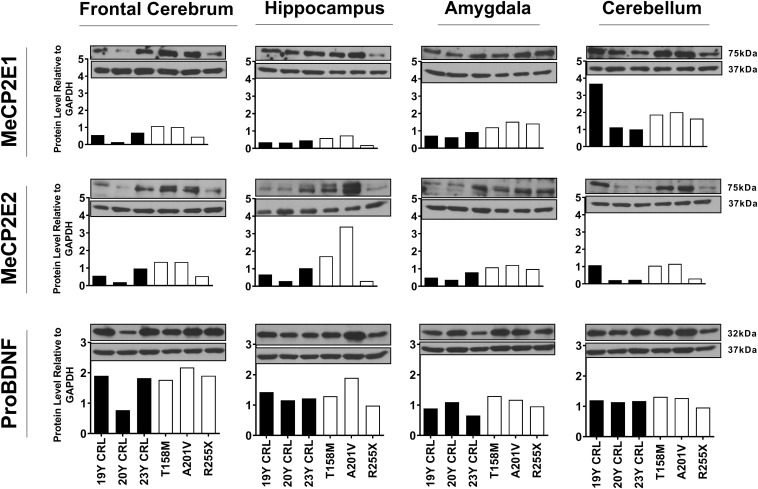
Western blot results of MeCP2E1, MeCP2E2, and ProBDNF in human Rett Syndrome (RTT) and control brain tissues. Western Blot results for MeCP2 isoforms and ProBDNF normalized to GAPDH in three RTT patients and their age-, and sex-matched controls show inter-regional variations as well as variable amount of proteins among controls and RTTs. The full blots for ProBDNF (including mature BDNF) with GAPDH loading control are provided in Supplementary Figure S1. Control (CRL); Year Old (Y).

**FIGURE 3:**
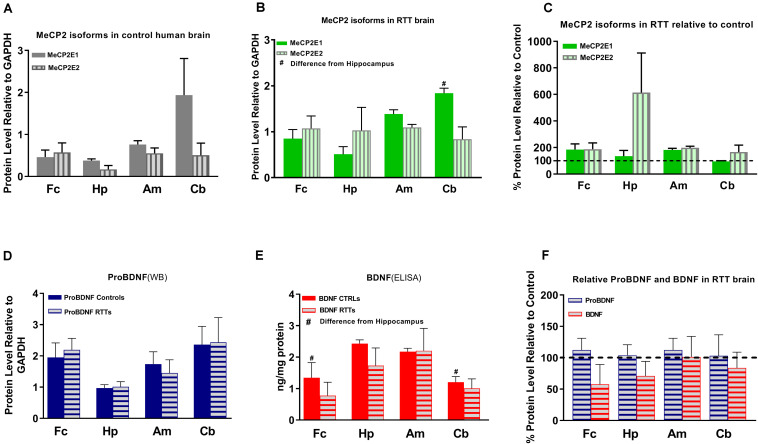
Results of protein studies by Western blot and ELISA in different regions of Rett Syndrome (RTT) and control brain tissues. **(A)** Western blot results for MeCP2 isoforms quantified and analyzed in four brain regions (frontal cerebrum, hippocampus, amygdala, and cerebellum) for mean of controls show non-significant variations between the regions. **(B)** Western blot results for MeCP2 isoforms quantified and analyzed in four brain regions for mean of RTTs show higher level of MeCP2E1 isoform in the cerebellum compared to the frontal cerebrum or hippocampus. **(C)** Mean of MeCP2 isoforms in RTT and controls are not significantly different in relative RTT/control analysis. **(D)** Western blot results for ProBDNF quantified and analyzed in four brain regions for mean of controls and RTTs show non-significant inter-regional variations without significant difference between RTT and controls. **(E)** ELISA results for BDNF quantified and analyzed in four brain regions for mean of controls and RTTs show significantly higher levels in Hippocampus compared to frontal cerebrum and cerebellum of the controls. **(F)** Mean of ProBDNF and BDNF in RTT and controls are not significantly different in relative RTT/Control analysis. Frontal cerebrum (Fc); Hippocampus (Hp); Amygdala (Am); Cerebellum (Cb); *N* = 3 ± Standard Error of the Mean (SEM); One-way ANOVA (ELISA)/Kruskal-Wallis test (WB) with multiple pairwise comparisons used among different brain regions. Student’s *t*-test (ELISA)/Mann-Whiney test (WB) was used to compare controls and RTT samples for each parameter. The significance level was determined at *P* < 0.05 (#).

Others and us have reported that MeCP2 protein and transcript levels do not always correlate with each other (Balmer et al., 2003; Mullaney et al., 2004; Liyanage et al., 2013; McGowan and Pang, 2015). Therefore, we next analyzed the protein levels of MeCP2E1 and MeCP2E2 in the control and RTT patient brain tissues. As MeCP2 is a nuclear protein, we isolated the nuclear and cytoplasmic extracts of frozen brain tissues of RTT and control brain samples, including the frontal cerebrum, hippocampus, amygdala, and cerebellum. WB experiments on isolated nuclear extracts with our validated custom-made anti-MeCP2E1 and MeCP2E2 antibodies (Zachariah et al., 2012; Olson et al., 2014), indicated that both MeCP2E1 and MeCP2E2 proteins are detectable in RTT and control brain tissues at 72–75 kDa (Figure 2). Despite consistent detection of GAPDH in different samples (indicating comparable loading from each sample), MeCP2 isoforms were inconsistently detected in both the controls and RTT samples. In this regard, the 20 year-old control brain tissues showed lower level of both MeCP2 isoforms in the frontal cerebrum. Also, MeCP2E2 showed relatively low levels in two out of the three controls in the majority of tested brain regions (Figure 2). The post-mortem delay (PMD) of these control samples (Figure 2) from left to right are as following: 22 h (NIH Neurobiobank #5401, 19 years old female), 23 h (NIH Neurobiobank #5646, 20 years old female), and 15 h (NIH Neurobiobank #5287, 23 years old female). In case of any impact of PMD on protein detection of MeCP2E1 and MCP2E2, it is possible that our results on MeCP2 protein levels may not properly reflect the actual intact protein level, and that the protein may have undergone some degradation. We recently reported that the highest intact detection of MeCP2 in surgical samples is associated with the 1 h time-window after sample collection (Pejhan et al., 2020), which may not be feasible to achieve in all cases of post-mortem organ donations. In line with the detected MeCP2 levels with high variations, most RTT brain regions also showed inconsistent levels. Of note, the two RTT brain tissues (T158M and A201V) that we received through organ donation (with PMD of <6 and 24 h, respectively) showed higher MeCP2E1 and MeCP2E2 levels compared to almost all other tested samples. The fact that the A201V sample with 24 h PMD still shows much higher MeCP2 protein levels compared to even control samples, highlights that MeCP2 homeostasis at the protein level might be more complicated as previously thought. As expected, the R255X non-sense mutation showed relatively low level of both MeCP2 isoforms (at 72–75 kDa), originated from the non-mutated X-chromosome. Non-parametric comparisons between the controls and RTT samples did not show any significant differences of the MeCP2 isoforms (Figures 3A–C), which was not in concordance with the results of their corresponding transcript levels.

### BDNF and ProBDNF Levels in the Brain of RTT Patients and Controls

Next, we investigated BDNF protein levels in these brain tissue samples. As BDNF is a cytoplasmic protein (Wetmore et al., 1991; Aliaga et al., 2009), we performed WB experiments with isolated cytoplasmic extracts. Our results showed the presence of the mature BDNF at around 14 kDa, and proBDNF at 32 kDa (Supplementary Figure S1). The detected bands were relatively consistent within the control group, with no obvious differences between the control and RTT brain tissues (Figures 2, 3D,E and Supplementary Figure S1). Surprisingly, we detected very low levels of the mature BDNF in the cerebellum of both RTT and control tissues (Supplementary Figure S1). To confirm our findings, we further studied BDNF levels using ELISA kits (Sigma-Aldrich), as a parallel complementary approach. Based on ELISA results, control hippocampus samples showed statistically significant higher levels of BDNF compared to the frontal cerebrum and cerebellum. The average level of BDNF in control hippocampus was 1.81 and 2.02 times higher than the frontal cerebrum and cerebellum, respectively (*P* < 0.05). Also, the amygdala and hippocampus showed higher levels of the mature BDNF compared to the other two brain regions. However, inter-regional differences among RTT samples were not statistically significant (Figure 3E). As comparing Western blot results in Figure 3D with ELISA results in Figure 3E shows, regions with higher mature BDNF showed lower levels of ProBDNF precursor, although the detected difference among these regions was not statistically significant. RTT samples had similar levels of proBDNF, with a trend of lower levels of mature BDNF in different regions of the brain, which was not statistically significant (Figure 3F).

### Correlation Studies

**FIGURE 4:**
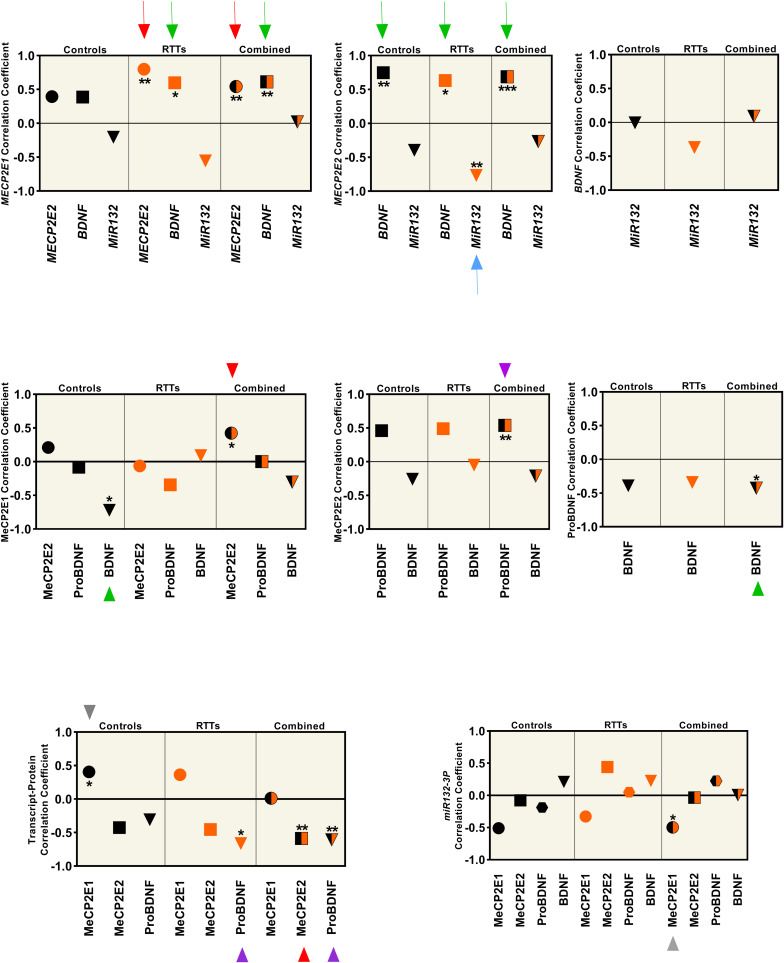
Correlation analysis between all the studied parameters (MeCP2 isoforms, ProBDNF, BDNF, and *miR132*) from four brain regions (Frontal Cerebrum, Hippocampus, Amygdala, and Cerebellum) in transcript and protein level. All graphs represent the Pearson’s (for parametric variables), or Spearman’s (for non-parametric variables) correlation coefficient in controls (black), RTTs (orange), and controls and RTTs combined (black/orange). Statistical significance: ***P < 0.001; **P < 0.01; *P < 0.05; The charts are showing the data collected from four different brain regions (Frontal Cerebrum, Hippocampus, Amygdala and Cerebellum) of 3 patients and their age and sex matched controls (*N* = 3 controls + 3 RTTs). The two isoforms are positively correlated at the transcript level, which is significant for RTT and mixed data (red arrow), and *MECP2E2* is negatively correlated with its protein, which is significant in mixed data (red arrowhead). Both isoforms show positive correlation with BDNF at the transcript level. Green arrows show the significant ones. BDNF protein has a significant negative correlation with MeCP2E1 in control samples (green arrowhead). ProBDNF is positively correlated with MeCP2E2, which is significant in mixed data (purple arrowhead). There is a negative correlation between ProBDNF and BDNF transcript in both RTT and mixed data (purple arrowhead). The only significant correlation of *miR132* is a negative correlation with *MECP2E2* in RTTs (blue arrow). Note that all *miR132* transcripts in this Figure refer to *miR132-3P* strand.

Next, we performed correlation of transcript-transcript and transcript-protein analysis among the studied parameters of RTT and control brain tissues (Figure 4).

We observed that the two *MECP2* isoforms were positively correlated at the transcript level (red arrows in Figure 4, top panel), which was statistically significant for RTT and combined data from controls and RTT samples together (*P* < 0.01). However, at the protein level, the two MeCP2 isoforms showed statistically significant positive correlation only for the combined data, which is marked by red arrowhead (*P* < 0.05) (Figure 4, middle panel). The transcript-protein correlation was negative for MeCP2E2, which was statistically significant for the combined data (*P* < 0.01), marked with red arrowhead in Figure 4 (bottom panel). The gray arrowhead in Figure 4 (bottom panel) points to positive transcript-protein correlation for MeCP2E1 for the control data (*P* < 0.05). Our studies showed that although both *MECP2* isoforms have a statistically significant positive correlation with *BDNF* at the transcript level (green arrow in Figure 4, top panel); however, at the protein level, BDNF is negatively correlated only with MeCP2E1 in control samples (green arrowhead in Figure 4, middle panel). ProBDNF on the other hand, showed a positive correlation only with MeCP2E2 in combined data (*P* < 0.01), which is marked by purple arrowhead in Figure 4, middle panel. Furthermore, we observed a negative correlation between BDNF and its precursor ProBDNF, which was statistically significant (*P* < 0.05) for combined data (green arrowhead in Figure 4, middle panel, right graph). ProBDNF also showed a negative correlation with *BDNF* transcripts, which was statistically significant for both RTT and combined data (purple arrowheads in Figure 4, bottom panel). Finally, *miR132* showed a negative correlation with *MECP2E2*, which was statistically significant (*P* < 0.01) in RTT brains (blue arrow in Figure 4, top panel).

### Histopathological Examination

**FIGURE 5:**
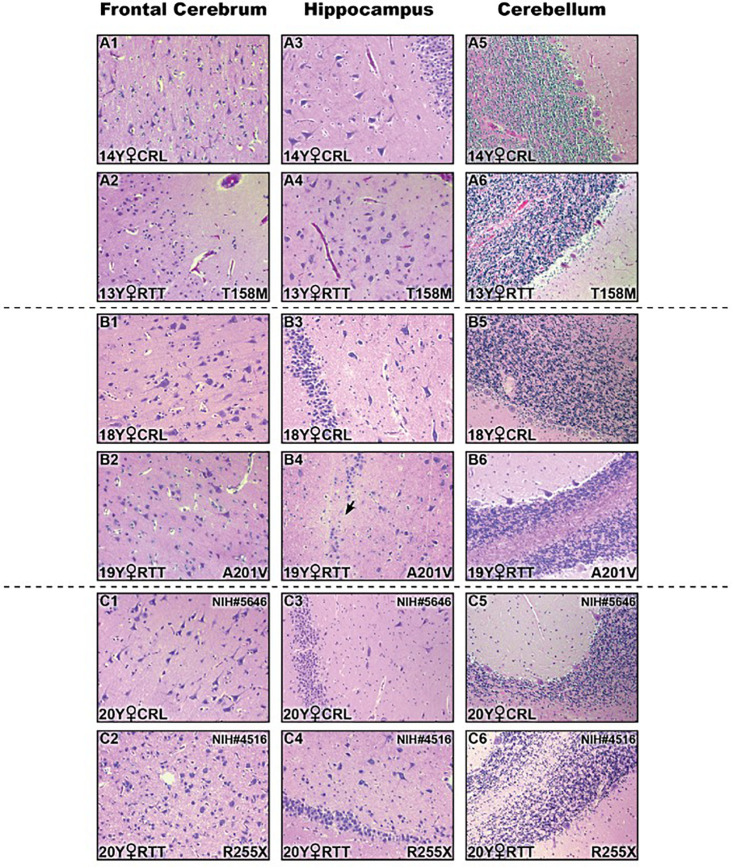
Histopathologic studies of different brain regions in three Rett Syndrome (RTT) patients and their sex-, and age-matched controls stained by Hematoxylin and Eosin (H&E). There were not noticeable findings at the microscopic levels. Two subtle changes are shown here: small pyramidal neurons and increased cell density in hippocampal region of T158M brain **(A4)** compared to the control **(A3)**; thin and hypocellular dentate gyrus in the hippocampus of A201V brain (black arrow in **B4**) compared to the control **(B3)**; Control (CRL); Year old (Y); 200× magnification.

In addition to molecular analysis of the RTT brain compared to controls, we then studied if these examined RTT brain tissues show any gross anatomical abnormalities at the pathological levels. Therefore, we performed histopathological investigation of the frontal cerebrum, hippocampus, and cerebellum in RTT patients and their sex- and age-matched controls by Hematoxylin and Eosin staining of the prepared slides (Figure 5). Formalin-fixed amygdala was not available for all samples, and was therefore removed from the H&E analysis. The microscopic evaluation was unremarkable without features of dead neurons or inflammation. Increased cell density and smaller pyramidal neurons in comparison with age- and sex-matched controls were observed in the hippocampus of RTT brain with the T158M mutation (Figure 5A4 versus A3). In RTT brain with A201V mutation, the dentate gyrus of the hippocampal formation was slightly thin toward the end and focally hypocellular. These subtle findings of microscopic examination are shown in Figure 5.

## Discussion

### Studies on Human Brain Tissues Support That Components of the MeCP2-BDNF-*miR132* Regulatory Loop Are Expressed in a Region-Dependent Manner in the Human Brain

Our finding to detect lower transcript levels of *MECP2* isoforms in the human RTT brain is in agreement with previous studies that reported decreased *MECP2* transcripts in Rett Syndrome (Chen et al., 2001; Petel-Galil et al., 2006). In our study, MeCP2E2, and ProBDNF showed a mainly negative correlation with their transcripts, which was opposite to what we saw for MeCP2E1. The correlation coefficient was not similar for RTT and controls in all cases (Figure 4). Complex regulation of protein expression in the human brain has been suggested as a possible reason behind the low predictive value of transcript expression for the corresponding protein(s) (Bauernfeind and Babbitt, 2017). Post-transcriptional control, and cell- or tissue-specific regulation rather than transcriptional-specific control are also among other possible explanations (Shahbazian et al., 2002a). In the human post-mortem RTT brain, *BDNF* transcripts showed a similar pattern to *MECP2E1/E2* with lower transcript expression, without any significant difference at the protein levels compared to controls. In this regard, our recent report on the formalin-fixed brain tissues added another layer to the complexity of MeCP2-BDNF expression. In this previous study, BDNF immunolabeling showed that different cells of the brain i.e., astrocytes, endothelial cells, and neurons could all be part of MeCP2 homeostasis (Pejhan et al., 2020). Therefore, it might be too simplistic to suggest a single regulatory mechanism without considering the cellular source of the proteins. Furthermore, the inter-regional variations that we showed in the elements of the suggested MeCP2 regulatory network in the human brain might be the result of variable number of cells and different cell types with different functions in each region, which makes finding a unique regulatory system in the whole brain of human less probable. Studying the third element of the MeCP2 homeostasis regulatory network proposed in rodents (*miR132*), a brain region-specific expression was identified specific to the cerebellum, where *MECP2E1-E2* and *BDNF* levels were the highest and *miR132* levels were found to be the lowest (Figure 1). Interestingly, cerebellum was the single brain region that both *miR132* strands were detected at similar levels between RTT patients and control samples. In general, lower transcript levels of *MECP2* isoforms in the human RTT brains were associated with lower level of *miR132* in all brain regions, except for the cerebellum. As indicated, cerebellum was different from the other three tested brain regions with similar level of *miR132* in RTT and control brains. Looking at an average of all brain regions in our correlation studies, we noticed a negative correlation between *miR132* and both *MECP2E1* and *MECP2E2* transcripts, which was significant for the latter (Figure 4). Previous *in vivo* studies in rodents have shown that increased level of *miR132* is associated with decreased level of MeCP2 and BDNF in the rat hippocampus (Su et al., 2015), suggesting the potential existence of species-specific regulatory mechanisms between rodents and humans. In the future, we will be interested to study the detailed mechanism of MeCP2 homeostasis regulation in human brain cells using relevant *in vitro* studies. Densely packed neurons with smaller soma size and reduced dendritic complexity are important morphological features of RTT brains compared to controls. These characteristics have been extensively studied in *in vitro* differentiated human neurons (Marchetto et al., 2010; Chin et al., 2016), and in rare studies on post-mortem brain samples of RTT patients (Armstrong et al., 1995). Although this was out of the scope of our current study; it would be important to investigate such changes in various type of neurons in different brain regions of RTT patients to establish if similar abnormalities are region, or cell type-specific in the human brain.

### ProBDNF and Mature BDNF in RTT and Control Human Brain Samples

Most studies on MeCP2-BDNF interactions in RTT pathophysiology have focused on neurons while our previous study showed a clear astroglial/endothelial pattern of immunostaining for BDNF in human brain samples (Pejhan et al., 2020). Apart from the BDNF source of expression, its impairment in RTT patients is another controversy. Here, we showed lower *BDNF* mRNA expression in RTT brain. However, BDNF protein levels (detected by WB, ELISA, and previously by IHC (Pejhan et al., 2020) were not detected at lower levels in the human RTT patient brain tissues, suggesting RTT brain has comparable BDNF levels to controls. We detected equal to higher BDNF levels in the Purkinje cells of the RTT cerebellar tissues by immunohistological studies (Pejhan et al., 2020). Such weak predictive value of transcript expression for their corresponding proteins is not unusual and has been linked to complex regulation of protein expression in the human brain (Bauernfeind and Babbitt, 2017). Of note anti-BDNF antibody can detect both proBDNF and the mature protein in ELISA and IHC. Only WB experiment differentiates between proBDNF and the mature BDNF by molecular size, but it also showed no difference between control and RTT brain. Our WB results with lower level of mature BDNF and higher level of ProBDNF in the cerebellum are in line with our previous findings with IHC (Pejhan et al., 2020). Furthermore, BDNF immunoreactivity has been shown to increase with conditions such as hypoxia or neuroinflammation (Hartman et al., 2015; Satriotomo et al., 2016), which are part of the pathogenesis of different neurological diseases, not fully studied in the context of RTT brain. Our correlation studies at the transcript level of *MECP2* isoforms and *BDNF* were in line with increased level of MeCP2 and BDNF as part of a regulatory effect, suggested in previous studies on rat neurons and rat brain (Klein et al., 2007; Su et al., 2015). However, at the protein level, MeCP2E1 mainly showed a negative correlation with BDNF, and for MeCP2E2, the positive correlation was shown mainly with proBDNF, and not the mature protein (Figure 4). While our data provide important insight about the components of MeCP2E1/E2-BDNF-*miR132* homeostasis regulatory network in the human brain and its impairment in RTT patients, additional studies are required to investigate the conservation of this regulatory network from rodents to humans.

## Limitations

Similar to previous studies that used post-mortem human brain tissues for Rett Syndrome, one limitation of our study was the absence of a large cohort of post-mortem RTT brain tissues. While RTT is a rare disease, we still obtained three independent biological replicates from different regions of the brain for human RTT patients and comparable controls, for statistical analysis of our results. Furthermore, our recent IHC examination of RTT human brain tissues (Pejhan et al., 2020) has shown the significance of cell-type variability for the elements studied in this project. Therefore, we acknowledge that studying the frozen brain lysate gives us an overall result for different cell types with potentially variable regulatory systems in a complex organ such as the human brain.

## Conclusion

Our findings from post-mortem human brain tissues suggest the existence of a brain region-specific regulation of the MeCP2E1/E2-BDNF-*miR132* that might be differently regulated in the cerebellum. Although, MeCP2 homeostasis regulation is still poorly understood, our data suggest that it is compromised in the human RTT brain, at least at the transcript levels. Our findings suggest that similar to other model systems, *MECP2E1* and *MECP2E2* transcript and protein may not be fully correlated in the human brain. In addition, BDNF transcript and protein levels do not show the same pattern of altered expression in the human RTT brain, with potential regulatory mechanisms that control BDNF maturation from its precursor ProBDNF protein. Despite intensive efforts in studying MeCP2 function in neurodevelopment, immune system (Pecorelli et al., 2020), and human cancer (Neupane et al., 2016), our findings would help to shed some light on its still obscure regulatory network. Several more common neurodevelopmental conditions such as fetal alcohol spectrum disorders or autism spectrum disorders with the evidence for the role of MeCP2 in their molecular biology of disease (Liyanage et al., 2015, 2017; Rastegar, 2017a; Amiri et al., 2020) may also benefit from the results of our study. Further *in vitro* studies could prove to be important for addressing whether MeCP2 homeostasis regulation is conserved from rodents to humans.

## Data Availability Statement

The raw data supporting the conclusions of this article are available by authors, upon request.

## Ethics Statement

Our research on human brain tissues was reviewed and approved by the University of Manitoba Bannatyne Campus Research Ethics Board with an approved Health Research Board protocol #HS20095 (H2016:337). Consent to perform research on donated human brain tissues was obtained from patient immediate family members.

## Author Contributions

SP and MR designed the experiments. SP performed the RT-PCR, WB, and ELISA. MD dissected the A201V and T158M human brain regions, and provided control tissues for H&E samples (Figures 5A,B). MR provided the conception and design, contributed reagents, materials, analysis tools, and research facilities. SP and MR wrote the manuscript. All authors read and approved the final version of the manuscript.

## Conflict of Interest

The authors declare that the research was conducted in the absence of any commercial or financial relationships that could be construed as a potential conflict of interest.

## Supplementary Material

FIGURE S1 Western blot analysis of ProBDNF and mature BDNF in Rett Syndrome (RTT) and control brain tissues. ProBDNF is detected around 32 kDa while the mature BDNF is detected around 14 kDa. The bands detected around 28 kDa have been observed by other groups and are suggested to be truncated protein (Mowla et al., 2001). Mature BDNF has a significantly lower level in the cerebellar tissues of controls and RTT tissues compared to other studied brain regions (frontal cerebrum, hippocampus, and amygdala). However, the level of ProBDNF in the cerebellum is comparable to other regions. Control (CRL); Year old (Y); Frontal cerebrum (Fc); Hippocampus (Hp); Amygdala (Am); Cerebellum (Cb); Kilodaton (kDa).

TABLE S1 List of primers used for qRT-PCR in this project.

TABLE S2 List of primary and secondary antibodies.

## Abbreviations

BDNF (protein)/*BDNF* (human gene): brain derived neurotrophic factor
ELISA: enzyme-linked immunosorbent assay
GAPDH (protein)/*GAPDH* (human gene): Glyceraldehyde 3-phosphate dehydrogenase
H&E: Hematoxylin and Eosin
IHC: immunohistochemistry
MeCP2 (protein)/*MECP2* (human gene)/*Mecp2* (mouse gene): Methyl CpG binding Protein 2
*miR132*: *microRNA132*
PMD: post-mortem delay
qRT-PCR: quantitative reverse transcription polymerase chain reaction
RTT: Rett syndrome
WB: Western blot.
