# Supplementary figures and images for: The MeCP2E1/E2-BDNF-miR132 Homeostasis Regulatory Network Is Region-Dependent in the Human Brain and Is Impaired in Rett Syndrome Patients
Source: Front Cell Dev Biol. 2020 Aug 21;8:763. doi: 10.3389/fcell.2020.00763 (PMC7471663; doi:10.3389/fcell.2020.00763)

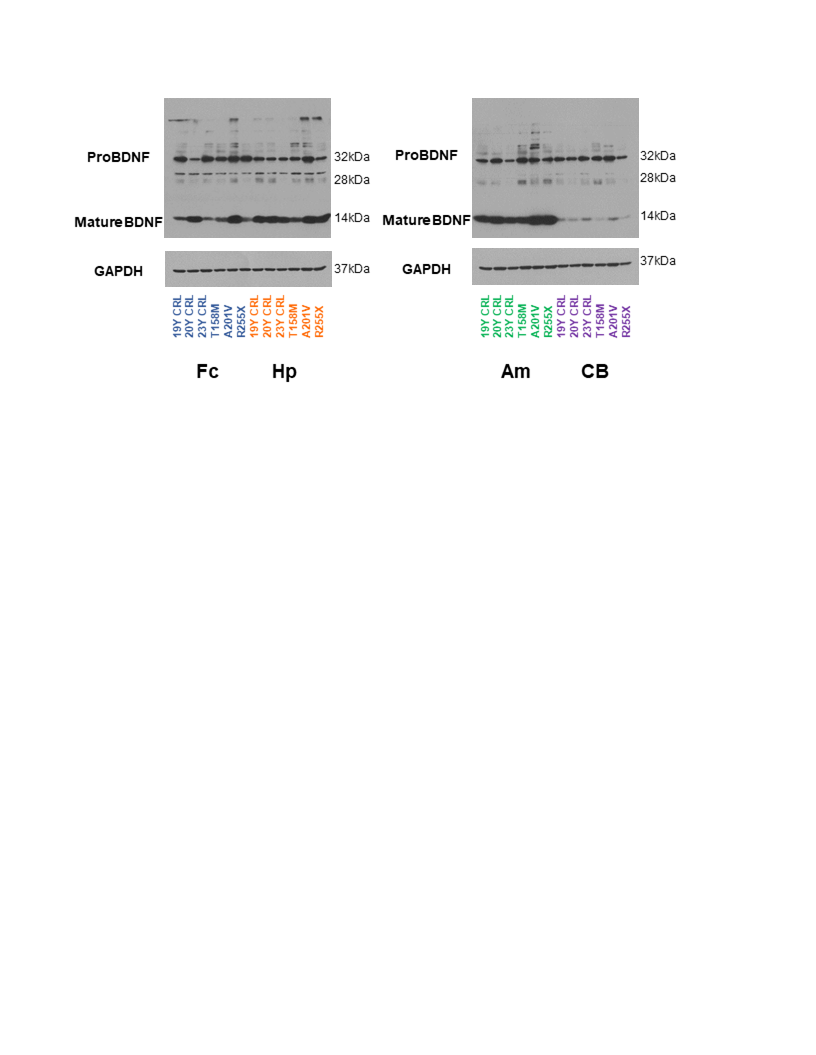

Supplement: FIGURE S1 — Western blot analysis of ProBDNF and mature BDNF in Rett Syndrome (RTT) and control brain tissues. ProBDNF is detected around 32 kDa while the mature BDNF is detected around 14 kDa. The bands detected around 28 kDa have been observed by other groups and are suggested to be truncated protein (Mowla et al., 2001). Mature BDNF has a significantly lower level in the cerebellar tissues of controls and RTT tissues compared to other studied brain regions (frontal cerebrum, hippocampus, and amygdala). However, the level of ProBDNF in the cerebellum is comparable to other regions. Control (CRL); Year old (Y); Frontal cerebrum (Fc); Hippocampus (Hp); Amygdala (Am); Cerebellum (Cb); Kilodaton (kDa). [file Image_1.TIF]
