# Supplementary material for: The MeCP2E1/E2-BDNF-miR132 Homeostasis Regulatory Network Is Region-Dependent in the Human Brain and Is Impaired in Rett Syndrome Patients
Source: Front Cell Dev Biol. 2020 Aug 21;8:763. doi: 10.3389/fcell.2020.00763 (PMC7471663; doi:10.3389/fcell.2020.00763)
Supplement: TABLE S1 — List of primers used for qRT-PCR in this project. [file Table_1.docx]

**Table S1.** **List of primers used for qRT-PCR in this study.**

| **Gene** | **Forward** | **Reverse** | **Reference** |
| --- | --- | --- | --- |
| *MECP2E1* | 5’-AGGAGAGACTGGAGGAAAAGTC-3’ | 5’-CTTGAGGGGTTTGTCCTTGA-3’ | (1) |
| *MECP2E2* | 5’-CTCACCAGTTCCTGCTTTGATGT-3’ | 5’-CTTGAGGGGTTTGTCCTTGA-3’ | (1) |
| *BDNF* | 5’-TAACGGCGGCAGACAAAAAGA-3’ | 5’-GAAGTATTGCTTCAGTTGGCCT-3’ | (2) |
| *GAPDH* | 5’-CCACTCCTCCACCTTTGAC-3’ | 5’-ACCCTGTTGCTGTAGCCA-3’ | (3) |

**Table S2. List of primary and secondary antibodies.**

| **Antibody** | **Application and Dilution** | **Description** | **Source** |
| --- | --- | --- | --- |
| BDNF  (within aa 150 to the C-terminus) | WB 1:500 | Rabbit monoclonal | Abcam, Ontario, Toronto, Canada,  ab108319 |
| BDNF  (within aa 128-247) | ELISA Capture and Detection | Mouse monoclonal | MilliporeSigma, Billerica, MA, USA, RAB0026 |
| GAPDH | WB 1:3000 | Rabbit polyclonal | Santa Cruz, Dallas, Texas, USA,  sc25778 (4) |
| MeCP2E1 | WB 2 μg/ml | Chicken Polyclonal | Custom-made (5, 6) |
| MeCP2E2 | WB 2 μg/ml | Chicken Polyclonal | Custom-made (6) |
| Peroxidase-AffiniPure donkey anti-rabbit IgG | WB 1:7500 | Secondary Ab | Jackson ImmunoResearch  711-035-152 |
| Peroxidase-AffiniPure Goat anti-chicken IgG | WB 1:7500 | Secondary Ab | Jackson ImmunoResearch  103-035-155 |

1. G. N. Mnatzakanian, H. Lohi, I. Munteanu, S. E. Alfred, T. Yamada, P. J. MacLeod, J. R. Jones, S. W. Scherer, N. C. Schanen, M. J. Friez, J. B. Vincent and B. A. Minassian: A previously unidentified MECP2 open reading frame defines a new protein isoform relevant to Rett syndrome. *Nat Genet*, 36(4), 339-41 (2004) doi:10.1038/ng1327

2. C. Zuccato, M. Marullo, B. Vitali, A. Tarditi, C. Mariotti, M. Valenza, N. Lahiri, E. J. Wild, J. Sassone, A. Ciammola, A. C. Bachoud-Levi, S. J. Tabrizi, S. Di Donato and E. Cattaneo: Brain-derived neurotrophic factor in patients with Huntington's disease. *PLoS One*, 6(8), e22966 (2011) doi:10.1371/journal.pone.0022966

3. L. Zou, Q. Chen, Z. Quanbeck, J. E. Bechtold and D. S. Kaufman: Angiogenic activity mediates bone repair from human pluripotent stem cell-derived osteogenic cells. *Sci Rep*, 6, 22868 (2016) doi:10.1038/srep22868

4. K. Sheikholeslami, A. Ali Sher, S. Lockman, D. Kroft, M. Ganjibakhsh, K. Nejati-Koshki, S. Shojaei, S. Ghavami and M. Rastegar: Simvastatin Induces Apoptosis in Medulloblastoma Brain Tumor Cells via Mevalonate Cascade Prenylation Substrates. *Cancers (Basel)*, 11(7) (2019) doi:10.3390/cancers11070994

5. R. M. Zachariah, C. O. Olson, C. Ezeonwuka and M. Rastegar: Novel MeCP2 isoform-specific antibody reveals the endogenous MeCP2E1 expression in murine brain, primary neurons and astrocytes. *PLoS One*, 7(11), e49763 (2012) doi:10.1371/journal.pone.0049763

6. C. O. Olson, R. M. Zachariah, C. D. Ezeonwuka, V. R. Liyanage and M. Rastegar: Brain region-specific expression of MeCP2 isoforms correlates with DNA methylation within Mecp2 regulatory elements. *PLoS One*, 9(3), e90645 (2014) doi:10.1371/journal.pone.0090645
